# Can a multitiered copayment system affect people’s healthcare-seeking behavior? A case study of Wenzhou, China

**DOI:** 10.1186/s12913-022-08031-0

**Published:** 2022-05-12

**Authors:** Lizheng Ge, Xiangyang Zhang, Yunyun Huang, Tingke Xu, Qianru Zhao, Tingting Zhu, Jingye Pan, Chun Chen

**Affiliations:** 1 School of Public Health and Management, Wenzhou Medical University, Zhejiang 325000 Wenzhou, China; 2 First Affiliated Hospital of Wenzhou Medical University, Zhejiang 325000 Wenzhou, China; 3 School of Innovation and Entrepreneurship, Wenzhou Medical University, Wenzhou, 325000 Zhejiang China; 4 Center for Health Assessment, Wenzhou Medical University, Wenzhou, 325000 Zhejiang China

**Keywords:** Multitiered copayment system, Primary healthcare-seeking behavior, Primary healthcare, Sustainable healthcare system, China

## Abstract

**Background:**

Facilitating the primary health care (PHC) system and maintaining people’s reasonable healthcare-seeking behavior are key to establishing a sustainable healthcare system. China has employed a multitiered copayment system/medical insurance differentiated payment policies to incentivize the public to utilize PHC services through its hierarchical medical care system; however, most people still prefer visiting tertiary care hospitals. We question whether the quality gap in healthcare services reduces the effect of the multitiered copayment system, which is considered an important factor in the lack of reform in the Chinese healthcare system. Thus, we explore the effect and influencing factors of the multitiered copayment system that drives primary healthcare-seeking behavior under the current situation with a large quality gap. We also consider the hypothetical situation of a reduced gap in the future.

**Methods:**

This study used the hypothetical quality improvement scenario to elicit people’s hypothetical behaviors, and a multistage stratified cluster random sampling method. This preliminary study was conducted in 2016 using 1829 individuals from four regions of Wenzhou in Zhejiang Province: Ouhai, Ruian, Yongjia, and Taishun. A descriptive statistical analysis, chi-square analysis, Fisher’s exact test, and multinomial logistic regression model were performed to introduce the effect of the multitiered copayment system, and to explore the factors affecting the selection of PHC institutions at pre- and post-change phases.

**Result:**

The results show that compared with the large quality gap phase, the number of respondents who believed the multitiered copayment system had an effect on their selection of PHC institutions after the equalization of healthcare services quality increased threefold (from 14.0% to 50.8%). Moreover, the main determinants in people’s selection of PHC institutions changed from age and needs variables (self-rated health status) to age, needs variables (self-rated health status) and enabling variables (distance to a medical care facility).

**Conclusion:**

The results indicate limited initial effects of the multitiered copayment system. However, they become more pronounced after the equalization of healthcare services quality. This study confirms that changes in the quality gap in healthcare services influence the effect of the multitiered copayment system. Hence, reducing this gap can help achieve the intended outcome of the tiered healthcare insurance schedule.

**Supplementary Information:**

The online version contains supplementary material available at 10.1186/s12913-022-08031-0.

## Background

With a series of healthcare system reforms, China is committed to establishing a sustainable healthcare system through strengthening its primary health care (PHC) system. One of the most challenging reforms to strengthen the PHC system has been establishing the hierarchical medical care system (HMS), announced in 2015 [1]. China has a three-tier healthcare system comprising tertiary, secondary, and primary hospitals [2, 3]. The HMS classifies diseases according to severity and treatment difficulty, and different tiers of hospitals provide different levels of healthcare services [1]. For example, PHC institutions mainly deal with common diseases, diagnosing and treating chronic diseases, minor surgery, health education, etc [4–6]. However, biased allocation of medical resources toward higher-tier hospitals, including sufficient medical facilities, adequate comprehensive technical support, and appropriate financial incentives [7], has resulted in a significant inconsistency in the quality of healthcare provided in China. Hence, patients in China prefer consulting doctors at secondary or tertiary tier hospitals regardless of the severity of their disease [3]. This situation persists despite crowded conditions, longer waiting time, and more significant expenses incurred at higher-tier hospitals. (The pricing policies for healthcare services in China allow higher-tier hospitals to charge more for the same services offered at lower-tier institutions [8]).

Therefore, promoting the HMS has become a significant problem in Chinese medical reform, which has hindered the establishment of a sustainable healthcare system. In recent years, China has resorted to using health insurance, including the multitiered copayment system/medical insurance differentiated payment policies, as the key policy to promote the HMS. The multitiered copayment system refers to reduce the copayment rates for patients visiting PHC institutions and higher-tier hospitals with a referral, but not for patients who can directly access the higher-tier hospitals without a referral [9]. This creates a system of differential deductible and copayment rates at different tiers of hospitals for inpatient and outpatient services, with the lowest copayment rate at the PHC institutions [10].

To date, the implementation of the multitiered copayment system has had little effect [11] as the higher-tier hospitals are still the first choice for most patients. In 2015, the number of visits to tertiary hospitals and PHC institutions was approximately 1.50 billion and 0.21 billion, respectively [12]. The number of visits to tertiary hospitals in 2019, however, was as high as 2.05 billion (37.7% increase compared to 2015), whereas at PHC institutions it was only 0.22 billion (11.7% increase compared to 2015) [12]. In 2019, there were approximately tenfold more visits to tertiary hospitals than to PHC institutions [12]. In 2020, tertiary hospital visits still exceeded primary institutions [12]. Statistically, the multitiered copayment system has not been successful in promoting people’s primary healthcare-seeking behavior (see Table 1).

**Table 1:** Number of visits in different tiers of hospitals from 2015 to 2020 (unit: billion)

Number of visits						
**Tiers**	**In 2015**	**In 2016**	**In 2017**	**In 2018**	**In 2019**	**In 2020**
Tertiary	1.497646	1.627848	1.726425	1.854787	2.057012	1.798245
Secondary	1.172331	1.216665	1.267851	1.284934	1.343425	1.156068
Primary	0.205679	0.217909	0.222173	0.224644	0.229652	0.202259

Source: China health statistical yearbook 2020 [12]

To establish a sustainable healthcare system, the Chinese government will continue implementing the multitiered copayment system. A growing body of research has confirmed that medical insurance to guide patients’ healthcare-seeking behavior is crucial to achieving HMS [13, 14]. Thus, it is essential to determine why this system has not changed people's healthcare-seeking behavior. Existing theoretical literature suggests that the healthcare service quality gap is a critical obstacle to Chinese medical reform [15], including the multitiered copayment system. In addition, empirical studies confirmed the limited effects of HMS [13] and multitiered copayment system [11] on patients’ healthcare-seeking behavior. However, few empirical studies have been done to verify whether the gaps in healthcare services quality hinder the beneficial effects of the multitiered copayment system.

Therefore, we conducted a preliminary study of the multitiered copayment system’s effects to analyze the influencing factors empirically. The study examines two situations, namely 1) the current situation of the large gap in the quality of healthcare services; and 2) the hypothetical situation of a reduced gap in the quality of healthcare services in the future.

## Materials and methods

### Data source

We collected the data from Wenzhou in 2016. Wenzhou was chosen because it was a pilot city to the implement of the HMS [16]. This status attached importance to the leverage of medical insurance in HMS. In addition, medical insurance policy practices in Wenzhou were standard throughout China. The copayment rate was the proportion that patients must pay after receiving health care services. The Urban Resident Basic Medical Insurance (URBMI) policy from Wenzhou in 2016 established a copayment rate for outpatient services of 50% at PHC institutions, 60% at secondary, and 75% at tertiary hospitals. For inpatient services, the PHC institution copayment rate was 10%. It was 20% at secondary and 25% at tertiary hospitals.

This study used a multistage stratified cluster random sampling method. First, four regions were selected from Wenzhou in the sample areas based on their levels of economic development: Ouhai, Ruian, Yongjia, and Taishun. Next as cross-sectional survey samples, four representative streets/towns were randomly selected from each region, and two committees/villages were randomly selected from each street/town. Then households in each committee/village were randomly selected by a table of random numbers from sampling distance. Finally, we sampled 30 households per community for our investigation.

The inclusion criteria for respondents were (1) individuals over the age of 15, (2) individuals who knew the details of the survey content and agreed to participate, and (3) individuals who were able to express their ideas clearly and voluntarily. The exclusion criteria for respondents were (1) individuals with visual impairment, and (2) individuals with delirium, dementia, and other mental disorders, and those with no interest in participating.

Face-to-face interviews were conducted to gather the data based on the multistage stratified cluster random sampling method. The survey questionnaire was divided into two dimensions: basic characteristics and the influence of the multitiered copayment system on healthcare-seeking behavior in current and hypothetical situations. Interviews were conducted with all family members in the selected households. (Following strategies of previous studies [17, 18], individuals over 15 years old who could understand the questionnaire and respond were chosen to guarantee survey reliability.) Finally, 960 households, 10 streets, and six towns were selected from the four regions for our sample. We distributed a total of 1,854 questionnaires, received 1,831 valid responses and deleted two missing values in this study. All data collected was cleaned and analyzed via EpiData and SPSS with double-checking.

### Dependent variables

The dependent variables included whether offering the multitiered copayment system would influence the public’s selection of PHC institutions in two situations, including 1) the current situation of the large gap in the quality of healthcare services and 2) the hypothetical situation of a reduced gap in the quality of healthcare services in the future.

The following questions were presented in the questionnaire: Does the current offering of the multitiered copayment system affect your selection of PHC institutions? Moreover, under the hypothetical situation of a reduced gap in the quality of healthcare services, will the offer of the multitiered copayment system still influence your selection of PHC institutions? The alternative responses were “have effect,” “no effect,” and “uncertainty.”

### Explanatory variables

#### Independent variables

The Anderson Model describes healthcare service use in behavioral terms [19]. It is a fully verified and recognized theoretical framework that aims to understand the determinants of healthcare service utilization [20]. The model emphasizes contextual and individual determinants [19]. According to the Anderson Model, independent variables were examined across three dimensions: predisposing, enabling, and need variables. Each dimension has different effects on access to healthcare services.

Predisposing variables: represent whether people’s healthcare needs and social status affect their healthcare-seeking behavior [19, 21, 22]. The predisposing variables in this study include age, education, marital status, and employment status.

Enabling variables: represent financial and social factors that affect people’s healthcare-seeking behavior [19]. The enabling variables in this paper included social support [23, 24], types of insurance [17, 23–25], distance to a medical care facility [24, 25], and household income [24, 25]. Social support was assessed by asking respondents if they could receive help from others (i.e., family, friends, colleagues, neighbors) when they needed it. There were three possible responses: “absolutely,” “occasionally,” and “not at all.” Based on participants’ responses, we classified their social support level as “good,” “medium,” or “poor.” Household income was reported based on 2015 values. As shown in Table 2, we distinguished four regions (Ouhai, Ruian, Taishun, Yongjia), and collapsed the data into quartiles for analysis (Q1/4 = “poor,” Q2/4 = “medium,” Q3/4 = “good”).

**Table 2:** Respondents’ household income in 2015 by region and quartile (unit: yuan)

Household Income			
**Region**	**Q1/4(Poor)**	**Q2/4(Medium)**	**Q3/4(Good)**
Ouhai	< 40,000	40,000–100,000	> 100,000
Ruian	< 50,000	50,000–130,000	> 130,000
Taishun	< 35,000	35,000–100,000	> 100,000
Yongjia	< 50,000	50,000–100,000	> 100,000

Good means a household’s income is high in their area; medium means a household’s income is medium in their area; poor means a household’s income is low in their area

Needs variables: represent people’s perception of their general health, functional status, and how the severity of their diseases affects their healthcare-seeking behavior [19]. The needs variables in this study include types of chronic diseases [17, 24] and self-rated health status [17, 23, 24]. The self-rated health status score was the respondents’ assessment of their health status on the day of the survey. The score ranged from 0 to 100. We classified total scores into quartiles [26]. (good: 91–100, medium: 70–90, poor: 0–69).

#### Time variable

It is generally accepted that a large gap exists in healthcare services between the higher-tier hospitals and PHC institutions in China. Thus, we defined the existing gaps in the quality of healthcare services between the higher-tier hospitals and PHC institutions as the pre-change phase. The hypothetical situation of a reduced gap in the quality of healthcare services in the future between the higher-tier hospitals and PHC institutions was defined as the post-change phase.

### Statistical analysis

This study used the hypothetical quality improvement scenario to elicit people’s hypothetical behaviors. We used SPSS version 26 to conduct the analysis. A descriptive statistical analysis was performed first to introduce the effect of the multitiered copayment system pre- and post-change. Next, a chi-square analysis and Fisher’s exact test were used to examine the differences between variables. The variables associated with *p* = 0.2 and below were entered into a multinomial logistic regression model to explore the factors affecting people’s selection of PHC institutions pre- and post-change [27].

## Results

### Basic characteristics

The study included 1829 respondents (51.4% men; average age 47.382 ± 14.945). In terms of education level, 59.9% had a primary or secondary school education, 13.3% had a high school or technical school education, and 10.8% had a college or higher education. Among enabling variables, the average household income was 97,914 ± 159,896 yuan. Most respondents (84.3%) lived within 10 min of a PHC institution, according to their usual mode of transportation. Among the need variables, the respondents’ average scores for self-rated health status were 78.929 ± 12.718 (Table 3).

**Table 3:** Characteristics of 1829 respondents in 2016

Variables	Characteristics	N(%)/MEAN ± SD(Range)	At pre-change phase						At post-change phase				
			**Have effects** **(%)**	**Uncertainty** **(%)**	**No effects** **(%)**	**χ**_2_		***P*-value**	**Have effects** **(%)**	**Uncertainty** **(%)**	**No effects** **(%)**	**χ**_2_	***P*-value**
**Predisposing variables**													
Gender	Male	941 (51.4)	128 (7.0)	22 (1.2)	791 (43.2)	0.395	0.821		480 (26.2)	131 (7.2)	330 (18.0)	0.056	0.973
	Female	888 (48.6)	128 (7.0)	23 (1.3)	737 (40.3)				450 (24.6)	122 (6.7)	316 (17.3)		
Age	47.382 ± 14.945 (15–93)												
Age group	15–24	86 (4.7)	21 (1.1)	5 (0.3)	60 (3.3)	49.688	< 0.001		46 (2.5)	21 (1.1)	19 (1.0)	65.102	< 0.001
	25–34	310 (16.9)	61 (3.3)	5 (0.3)	244 (13.3)				188 (10.3)	45 (2.5)	77 (4.2)		
	35–44	436 (23.8)	59 (3.2)	21 (1.1)	356 (19.5)				223 (12.2)	66 (3.6)	147 (8.0)		
	45–54	421 (23.0)	65 (3.6)	8 (0.4)	348 (19.0)				230 (12.6)	57 (3.1)	134 (7.3)		
	55–64	310 (16.9)	28 (1.5)	3 (0.2)	279 (15.3)				138 (7.5)	35 (1.9)	137 (7.5)		
	> = 65	266 (14.5)	22 (1.2)	3 (0.2)	241 (13.2)				105 (5.7)	29 (1.6)	132 (7.2)		
Education	No education	292 (16.0)	38 (2.1)	7 (0.4)	247 (13.5)	9.883	0.262		123 (6.7)	43 (2.4)	126 (6.9)	42.479	< 0.001
	Primary school	511 (27.9)	66 (3.6)	8 (0.4)	437 (23.9)				260 (14.2)	50 (2.7)	201 (11.0)		
	Secondary school	585 (32.0)	80 (4.4)	15 (0.8)	490 (26.8)				330 (18.0)	70 (3.8)	185 (10.1)		
	High school or technical school	244 (13.3)	40 (2.2)	5 (0.3)	199 (10.9)				120 (6.6)	51 (2.8)	73 (4.0)		
	College or higher	197 (10.8)	32 (1.7)	10 (0.5)	155 (8.5)				97 (5.3)	39 (2.1)	61 (3.3)		
Marital status	Married	1659 (90.7)	220 (12.0)	39 (2.1)	1400 (76.5)	9.021	0.009		845 (46.2)	210 (11.5)	604 (33.0)	23.803	< 0.001
	Other (Single, divorced, widowed and separated)	170 (9.3)	36 (2.0)	6 (0.3)	128 (7.0)				85 (4.6)	43 (2.4)	42(2.3)		
Employment status	Employment	1211 (66.2)	189 (10.3)	34 (1.9)	988 (54.0)	22.073	0.004		645 (35.3)	169 (9.2)	397(21.7)	55.366	< 0.001
	Unemployment	355 (19.4)	31 (1.7)	7 (0.4)	317 (17.3)				175 (9.6)	54 (3.0)	126(6.9)		
	Retired	215 (11.8)	23 (1.3)	4 (0.2)	188 (10.3)				84 (4.6)	15 (0.8)	116(6.3)		
	Student	43 (2.4)	12 (0.7)	0 (0.0)	31 (1.7)				24 (1.3)	14 (0.8)	5(0.3)		
	Others	5 (0.3)	1 (0.1)	0 (0.0)	4 (0.2)				2 (0.1)	1 (0.1)	2(0.1)		
**Enabling variables**													
Social support	Good	1304 (71.3)	183 (10.0)	26 (1.4)	1095 (59.9)	12.484	0.011		692 (37.8)	145 (7.9)	467 (25.5)	48.449	< 0.001
	Medium	449 (24.5)	68 (3.7)	19 (1.0)	362 (19.8)				202 (11.0)	104 (5.7)	143 (7.8)		
	Poor	76 (4.2)	5 (0.3)	0 (0.0)	71 (3.9)				36 (2.0)	4 (0.2)	36 (2.0)		
Types of insurance	UEBMI	249 (13.6)	36 (2.0)	13 (0.7)	200 (10.9)	15.855	0.010		117 (6.4)	41 (2.2)	91 (5.0)	6.171	0.404
	URBMI	1324 (72.4)	178 (9.7)	31 (1.7)	1115 (61.0)				678 (37.1)	186 (10.2)	460 (25.2)		
	NRCMI	208 (11.4)	36 (2.0)	0 (0.0)	172 (9.4)				111 (6.1)	19 (1.0)	78 (4.3)		
	No insurance	48 (2.6)	6 (0.3)	1 (0.1)	41 (2.2)				24 (1.3)	7 (0.4)	17 (0.9)		
Distance to a medical care facility	Arrive within 10 min (inclusive)	1541 (84.3)	220 (12.0)	40 (2.2)	1281 (70.0)	5.056	0.241		718 (39.3)	218 (11.9)	605 (33.1)	107.316	< 0.001
	Arrive within 20 min (inclusive)	277 (15.1)	36 (2.0)	4 (0.2)	237 (13.0)				211 (11.5)	32 (1.7)	34 (1.9)		
	Arrive more than 20 min	11 (0.6)	0 (0.0)	1 (0.1)	10 (0.5)				1 (0.1)	3 (0.2)	7 (0.4)		
Household Income	97,914 ± 159,896 (0–5,000,000)												
Household Income	Good	394 (21.5)	61 (3.3)	11 (0.6)	322 (17.6)	2.186	0.702		187 (10.2)	65 (3.6)	142 (7.8)	11.544	0.021
	Medium	1057 (57.8)	147 (8.0)	27 (1.5)	883 (48.3)				570 (31.2)	129 (7.1)	358 (19.6)		
	Poor	378 (20.7)	48 (2.6)	7 (0.4)	323 (17.7)				173 (9.5)	59 (3.2)	146 (8.0)		
**Need variables**													
Types of chronic diseases	0	1131 (61.8)	175 (9.6)	26 (1.4)	930 (50.8)	13.860	0.142		574 (31.4)	167 (9.1)	390 (21.3)	9.231	0.479
	1	459 (25.1)	55 (3.0)	13 (0.7)	391 (21.4)				242 (13.2)	60 (3.3)	157 (8.6)		
	2	175 (9.6)	16 (0.9)	6 (0.3)	153 (8.4)				85 (4.6)	16 (0.9)	74 (4.0)		
	3	50 (2.7)	6 (0.3)	0 (0.0)	44 (2.4)				22 (1.2)	8 (0.4)	20 (1.1)		
	4	10 (0.5)	2 (0.1)	0 (0.0)	8 (0.4)				5 (0.3)	1 (0.1)	4 (0.2)		
	> = 5	4 (0.2)	2 (0.1)	0 (0.0)	2 (0.1)				2 (0.1)	1 (0.1)	1 (0.1)		
Self-rated health status scores	78.929 ± 12.718 (1–100)												
Self-rated health status	Good	153 (8.4)	27 (1.5)	8 (0.4)	118 (6.5)	18.576	< 0.001		77 (4.2)	24 (1.3)	52 (2.8)	9.376	0.052
	Medium	1442 (78.8)	185 (10.1)	27 (1.5)	1230 (67.2)				713 (39.0)	201 (11.0)	528 (28.9)		
	Poor	234 (12.8)	44 (2.4)	10 (0.5)	180 (9.8)				140 (7.7)	28 (1.5)	66 (3.6)		

*UEBMI* Urban Employee Basic Medical Insurance, *URBMI* Urban Resident’s Basic Medical Insurance, *NRCMI* New Rural Cooperative Medical Insurance

### Effect of the multitiered copayment system on respondents’ primary healthcare-seeking behavior at pre- and post-change phases

In terms of the effect of the multitiered copayment system, at the pre-change phase, among respondents who thought the multitiered copayment system was effective, the proportion of those aged 15–24 years was 1.1%; 25–34 years, 3.3%; 35–44 years, 3.2%; 45–54 years, 3.6%; 54–64 years, 1.5%; and over 65 years, 1.2%. Among the self-rated health status, the proportion of those who were considered good was 1.5%; medium, 10.1%; and poor, 2.4%. Both age variable and self-rated health status variable were statistically significant (*p* < 0.001). In addition, the variables of marital status, employment status, social support, types of insurance all had statistical significance (*p* < 0.05) (Table 3).

At the post-change phase, among the respondents who thought the policy was effective, the proportion of those aged 15–24 years increased to 2.5%; 25–34 years, to 10.3%; 35–44 years, to 12.2%; 45–54 years, to 12.6%; 54–64 years, to 7.5%; and over 65 years, to 5.7%, with statistically significant differences (*p* < 0.001). Moreover, the variables of education, marital status, employment status and social support were all statistically significant (*p* < 0.001). Regarding the variable of distance to a medical care facility, 39.3% of respondents could reach a PHC institution within 10 min, and 11.5% within 20 min, with statistically significant differences (*p* < 0.001). The variable of household income was statistically significant (*p* < 0.05). At this phase, there was an increase in the number of respondents who believed that the multitiered copayment system had an effect on their selection of PHC institutions (Table 3).

### Factor analysis of respondents’ primary care-seeking behavior at pre- and post-change phases

Table 4 presents the results of the multinomial logistic regression analysis, which analyzed the factors that affect respondents’ selection of PHC institutions pre- and post-change. Respondents who considered that narrowing the gap in the quality of healthcare services between PHC institutions and the higher-tier hospitals would lead to a change in their primary healthcare-seeking behavior were used as the comparator group. The effect of the multitiered copayment system is reflected in the respondents’ selections of PHC institutions.

**Table 4:** Multinomial logistic regression: Factors affecting people’s healthcare-seeking behavior at pre- and post-change phases

Variables	Categories	No effect (Model 1) Comparator group = Have effects		No effect (Model2) Comparator group = Have effects	
		**OR (95% CI)**	***p*-value**	**OR (95% CI)**	***p*-value**
**Intercept**			**0.538**		**0.020**
**Predisposing variables**					
Age (Ref = > = 65)	15–24	2.867 (1.071–7.678)	0.036	1.677 (0.753–3.734)	0.206
	25–34	2.631 (1.406–4.925)	0.002	2.655 (1.655–4.260)	< 0.001
	35–44	1.792 (0.962–3.336)	0.066	1.629 (1.075–2.468)	0.021
	45–54	2.116 (1.170–3.825)	0.013	1.941 (1.314–2.867)	0.001
	54–64	1.190 (0.648–2.186)	0.574	1.354 (0.935–1.962)	0.109
**Need variables**					
Self-rated health status (Ref = Poor)	Good	0.654 (0.368–1.163)	0.148	0.620 (0.381–1.009)	0.054
	Moderate	0.486 (0.326–0.723)	< 0.001	0.571 (0.408–0.798)	0.001
**Enabling variables**					
Distance to a medical care facility (Ref = Arrive more than 20 min)	Arrive within 10 min (inclusive)	—	—	7.804 (0.942–64.618)	0.057
	Arrive within 20 min (inclusive)	—	—	39.243 (4.608–334.214)	0.001

Model 1 means at pre-change phase

Model 2 means at post-change phase

At the pre-change phase, the results indicated that age and self-rated health status were the main influencing factors. Compared with the respondents over 65 years, younger respondents (age group of 15–24, 25–34 and 45–54) who selected PHC institutions were less vulnerable to being affected by policy. It is evident that older respondents are typically more likely to be affected by the multitiered copayment system when compared to younger respondents. In addition, compared to respondents with poor self-rated health status, respondents with moderate self-rated health status are more sensitive to the incentive of the multitiered copayment system (Table 4).

At the post-change phase, age, self-rated health status and the enabling factor of distance to a medical care facility are the main influencing factors. Respondents in the age ranges of 24–34, 35–44, and 45–54 are not as susceptible to the multitiered copayment system as those over 65 years, and it also shows a trend in the pre-change phase. And compared to respondents with poor self-rated health status, respondents with moderate self-rated health status are more sensitive to being affected by policy, which was similar at the pre-change phase as well. In addition, compared with those who lived more than 20 min from a PHC institution, respondents who lived within 20 min were less susceptible to the multitiered copayment system (Table 4).

## Discussion

The present study examined the effects of the multitiered copayment system, and found that its introduction had different effects at the pre- and post-change phases. Compared with the pre-change phase, the number of respondents who believed the multitiered copayment system had an effect on their selection of PHC institutions at the post-change phase increased threefold (from 14.0% to 50.8%), and the multitiered copayment system started to play a positive role.

In the pre-change phase, owing to significant resource gaps, PHC institutions offered suboptimal quality healthcare services for common diseases [28]. The inconsistent service quality originated from many factors, including a shortage of medical facilities, inadequate comprehensive technical support, and inappropriate financial incentives in PHC institutions [7]. In particular, there was a low barrier to patient entry to higher-tier hospitals because a referral is not required [10]. In addition, related to a small gap in the copayment rate in different tiers, the reduction’s effect in the multitiered copayment rate on primary healthcare-seeking behavior was limited. This result corresponds with the results from a numerical experiment which concluded that in order to affect people’s primary healthcare-seeking behavior, the optimal copayment difference should be sufficiently large, such as 37% in tertiary hospitals and 4% in PHC institutions [11]. However, such a gap in the copayment rate would increase health and healthcare inequity. It was a challenge to increase the effectiveness of the multitiered copayment system by adjusting the copayment rate at the pre-change phase. Therefore, the multitiered copayment system failed to show a meaningful effect under the large quality gap between different tiers of healthcare services.

At the pre-change phase, the results of the multinomial logistic regression analysis indicate that the respondents’ age and the needs factor of self-rated health status are the main determinants of their selections of PHC institutions. Furthermore, these factors have also been proven in previous studies [29]. Age has been identified as the most common variable, which is similar in our study as well. We found that older people are more likely to change their primary healthcare-seeking behavior due to the copayment rate reduction. This may be because older adults are most likely to have chronic diseases that need continuous care [30], and the diagnosis and treatment of chronic diseases are highly standardized. In other words, the services required by older adults do not usually require advanced technology and equipment. Meanwhile, people with chronic conditions often report moderate or poor self-rated health status [30], which means there is a sizable overlap between the elderly and those with moderate and poor self-rated health status [31]. However, those with poor self-rated health scores tend to believe they have more complicated diseases, need higher levels of treatment, and that the services provided by PHC institutions cannot meet their medical care needs. Thus, due to their higher frequency of hospital visits and lower needs in diagnosis and treatment, the elderly and those with moderate self-rated health status would be motivated by the copayment rate reduction at PHC institutions.

As mentioned above, the effect of the multitiered copayment system will improve in the post-change phase. The role of the gap in the quality of healthcare services at PHC institutions was found in South Korea (non-equalization in services quality) and the United Kingdom (UK) (equalization in services quality). In the UK, the general practitioner system is the focal point of PHC [32], and the National Health Service attaches great importance to the quality of its healthcare services [33]. Thus, the key to system effectiveness is for general practitioners to provide consistent high quality healthcare services [34]. However, in Korea, a copayment policy was implemented to guide patients with mild diseases to PHC institutions [35]. The copayment rates for outpatients were 30% in primary care clinics and pharmacies, 40% in secondary hospitals, 50% or 60% in tertiary general hospitals [36]. However, the result of this copayment policy in Korea had little effect [37, 38], consistent with our findings as well as those from other studies in China. The quality of healthcare services in Korea was similar to that in China. Large gaps also existed between the higher-tier hospitals and PHC institutions, and allocation of medical care resources also tended to be biased towards higher-tier hospitals. Similarly, research has shown that the gatekeeping policy in China was counterproductive because of the weak PHC capacity [39, 40]. Thus, patients considered physicians in tertiary hospitals to be more qualified and preferred to choose these highly qualified physicians for healthcare, which resulted in the unsatisfactory effect from the multitiered copayment system [38]. All of this evidence indicates that the gap in quality of healthcare services was the key factor that determines the effectiveness of the multitiered copayment system.

At the post-change phase, age, needs factor of self-rated health status and the enabling factor of distance to a medical care facility were the critical factors affecting people’s selection of PHC institutions. At this phase, people no longer consider the gap in healthcare services quality, because they would receive the same quality of treatment regardless of the type of hospital visited. Thus, convenience of healthcare access is the primary factor to consider. In general, PHC institutions are closer to people’s homes, which makes it more convenient to receive healthcare services. Additionally, PHC institutions cover border, thus reduced patients’ waiting time, and always located in residential areas, thus providers there may be more familiar with patients living nearby, which makes it easier for these patients to receive targeted healthcare services [41].

## Limitations

This preliminary study has the following limitations. First, our samples are limited to Wenzhou, which is a medium-sized city in Zhejiang Province, southeastern China. Second, the results were based on respondents’ subjective answers to our questionnaire. Thus, the study scope needs to be expanded in the future, and future research should develop a more specific, objective tool to better measure the effect of the multitiered copayment system so that local governments can obtain more accurate information, adjust measures according to particular local conditions, and achieve optimal outcomes from the policy. Finally, future research should also examine the public’s willingness to pay for healthcare.

## Conclusions

The multitiered copayment system in Wenzhou has had little effect so far. Overall, we found that older adults and those with moderate self-rated health status are the main groups that are more likely to be affected by this policy. The low quality of care in PHC institutions in Wenzhou reduced the effectiveness of the multitiered copayment schedule’s intended outcome. In the context of the equalized quality of healthcare services in all tiers of healthcare facilities, the effect of the multitiered copayment system can be enhanced. Considering the factors of higher cost, long waiting time, and farther distance from home, seeking care at higher-tier hospitals is not the best choice for most people. Therefore, narrowing the gap in the quality of healthcare services among different tiers of hospitals is crucial in Wenzhou, as well as in other areas of China. In order to promote a sustainable healthcare system, more PHC healthcare institutions should be established at the community level. Meanwhile, the key aspect of creating an effective multitiered copayment system remains the improvement of the quality of healthcare services.

## Supplementary Information

**Additional file 1.** 

## Data Availability

All data used and analysed during this study are included in this published article and its supplementary information files.

## Abbreviations

PHC: Primary Health Care
HMS: Hierarchical medical care system
